# Nanotechnology for Pain Management

**DOI:** 10.3390/jcm13092611

**Published:** 2024-04-29

**Authors:** Jacques E. Chelly, Shiv K. Goel, Jeremy Kearns, Orkun Kopac, Senthilkumar Sadhasivam

**Affiliations:** 1 Department of Anesthesiology and Perioperative Medicine, University of Pittsburgh Medical Center, Pittsburgh, PA 15219, USA; goelsk@upmc.edu (S.K.G.); kearnsjt@upmc.edu (J.K.); kopaco@upmc.edu (O.K.); sadhasivams@upmc.edu (S.S.); 2 Department of Orthopaedic Surgery, University of Pittsburgh Medical Center, Pittsburgh, PA 15219, USA

**Keywords:** nanotechnology, nanotransporters, nanoparticles, micelles, quantum dots, liposomes, nanofibers, nano-scaffolds, nanocapacitors, acute pain, chronic pain, opioids

## Abstract

**Introduction**: In the context of the current opioid crisis, non-pharmacologic approaches to pain management have been considered important alternatives to the use of opioids or analgesics. Advancements in nano and quantum technology have led to the development of several nanotransporters, including nanoparticles, micelles, quantum dots, liposomes, nanofibers, and nano-scaffolds. These modes of nanotransporters have led to the development of new drug formulations. In pain medicine, new liposome formulations led to the development of DepoFoam™ introduced by Pacira Pharmaceutical, Inc. (Parsippany, NJ, USA). This formulation is the base of DepoDur™, which comprises a combination of liposomes and extended-release morphine, and Exparel™, which comprises a combination of liposomes and extended-release bupivacaine. In 2021, Heron Therapeutics (San Diego, CA, USA) created Zynrelef™, a mixture of bupivacaine and meloxicam. Advancements in nanotechnology have led to the development of devices/patches containing millions of nanocapacitors. Data suggest that these nanotechnology-based devices/patches reduce acute and chronic pain. **Methods**: Google and PubMed searches were conducted to identify studies, case reports, and reviews of medical nanotechnology applications with a special focus on acute and chronic pain. This search was based on the use of keywords like nanotechnology, nano and quantum technology, nanoparticles, micelles, quantum dots, liposomes, nanofibers, nano-scaffolds, acute and chronic pain, and analgesics. This review focuses on the role of nanotechnology in acute and chronic pain. **Results**: (1) Nanotechnology-based transporters. DepoDur™, administered epidurally in 15, 20, or 25 mg single doses, has been demonstrated to produce significant analgesia lasting up to 48 h. Exparel™ is infiltrated at the surgical site at the recommended dose of 106 mg for bunionectomy, 266 mg for hemorrhoidectomy, 133 mg for shoulder surgery, and 266 mg for total knee arthroplasty (TKA). Exparel™ is also approved for peripheral nerve blocks, including interscalene, sciatic at the popliteal fossa, and adductor canal blocks. The injection of Exparel™ is usually preceded by an injection of plain bupivacaine to initiate analgesia before bupivacaine is released in enough quantity from the depofoarm to be pharmacodynamically effective. Finally, Zynrelef™ is applied at the surgical site during closure. It was initially approved for open inguinal hernia, abdominal surgery requiring a small-to-medium incision, foot surgery, and TKA. (2) Nanotechnology-based devices/patches. Two studies support the use of nanocapacitor-based devices/patches for the management of acute and chronic pain. A randomized study conducted on patients undergoing unilateral primary total knee (TKA) and total hip arthroplasty (THA) provided insight into the potential value of nanocapacitor-based technology for the control of postoperative acute pain. The results were based on 2 studies, one observational and one randomized. The observational study was conducted in 128 patients experiencing chronic pain for at least one year. This study suggested that compared to baseline, the application of a nanocapacitor-based Kailo™ pain relief patch on the pain site for 30 days led to a time-dependent decrease in pain and analgesic use and an increase in well-being. The randomized study compared the effects of standard of care treatment to those of the same standard of care approach plus the use of two nanocapacitor-based device/patches (NeuroCuple™ device) placed in the recovery room and kept in place for three days. The study demonstrated that the use of the two NeuroCuple™ devices was associated with a 41% reduction in pain at rest and a 52% decrease in the number of opioid refills requested by patients over the first 30 days after discharge from the hospital. **Discussion**: For the management of pain, the use of nano-based technology has led to the development of nano transporters, especially focus on the use of liposome and nanocapacitors. The use of liposome led to the development of DepoDur™, bupivacaine Exparel™ and a mixture of bupivacaine and meloxicam (Zynrelef™) and more recently lidocaine liposome formulation. In these cases, the technology is used to prolong the duration of action of drugs included in the preparation. Another indication of nanotechnology is the development of nanocapacitor device or patches. Although, data obtained with the use of nanocapacitors are still limited, evidence suggests that the use of nanocapacitors devices/patches may be interesting for the treatment of both acute and chronic pain, since the studies conducted with the NeuroCuple™ device and the based Kailo™ pain relief patch were not placebo-controlled, it is clear that additional placebo studies are required to confirm these preliminary results. Therefore, the development of a placebo devices/patches is necessary. **Conclusions**: Increasing evidence supports the concept that nanotechnology may represent a valuable tool as a drug transporter including liposomes and as a nanocapacitor-based device/patch to reduce or even eliminate the use of opioids in surgical patients. However, more studies are required to confirm this concept, especially with the use of nanotechnology incorporated in devices/patches.

## 1. Introduction

The use of opioids to manage pain is an established determinant for the development of opioid use disorder in medical and surgical patients and therefore an important contributor to the current opioid crisis. Multiple factors are responsible for the present opioid epidemic and associated rate of death by overdose. The risk factors expressed as odds ratios are 1.75 for a history of addiction to alcohol [1,2,3,4,5,6], 1.94 for a history of addiction to tobacco/nicotine use [1,2,3,4,5,7,8,9,10,11,12], 1.59 for a history of depression [1,2,3,4,5,6,9,13,14,15,16,17], 1.67 for male gender [6,18], 2.35 for a prior/current history of substance abuse [19], and 1.62 for younger age [1,2,9,10,16,17,18,19,20,21,22]. Following surgery, the overall frequency of persistent opioid use is estimated to be 28% at two months [14], 9.96% at three months [1,2,3,4,10,11,15,16,20,23,24,25,26,27,28,29], 3.92% at six months [1,2,16,25], 2.82% at one year [1,2,6,12,15,30,31,32,33,34], and 1.85% at two years after surgery [17,18]. **After minor or ambulatory surgeries**, the overall estimated frequency of persistent opioid use is 7.5% at three months [4,10,29] and 7.7% at one year [30]. Among minor surgeries, elective hand surgery is associated with the highest frequency of persistent opioid use—13.5% at three months [10]. **After major surgeries**, the overall frequency of persistent opioid use is estimated to be 28% at two months [14], 10.4% at three months [1,2,3,4,11,15,16,20,23,25,26,27,28], 3.92% at six months [1,2,16,25], 2.43% at one year [1,2,12,15,31,32,33,34], and 1.85% at two years [17,18]. The major surgeries associated with the highest frequency of persistent opioid use are orthopedic surgeries, including joint replacement, trauma surgery, and surgery associated with cancer. For **orthopedic surgeries,** the overall frequency of persistent opioid use is estimated to be up to 28% at two months [14], the mean at three months is 9.79% [1,2,10,15,16,23,24,25], 3.92% at six months [1,2,16,25], 2.16% at one year [1,2,31,34], and 1.85% at two years [17,18]. More specifically, the frequency of persistent opioid use following **total knee arthroplasty** is estimated to be 13.4% at three months [2,25], 8.2% at six months [25], 1.8% at one year [34], and 1.4% at two years [18]. The frequencies are 7.8% at three months [1,15,25], 4.3% at six months [25], and 2.3% at two years [19] following **total hip arthroplasty.** For **oncology surgery**, it is estimated to be 17.53% at three months [11,28,29]; the estimated frequency seems to vary according to the type of surgery. It is 23.7% at three months for surgery for oral cavity cancer [11] and 18.5% at three months for head and neck oncology surgery [28]. For **trauma surgery**, the estimated frequency of persistent opioid use is estimated to be 28% at two months [14] and 11.35% at three months [10,23]. Several other types of surgeries have been investigated, including kidney transplantation (8.4% at one year [32]), bariatric surgery (1.3% at one year [12]), caesarean section (C-section) (0.36% at one year [9]), and dental surgery (0.1% at three months [29]). However, a recent report indicated that opioid use disorder increases after delivery. Aside from the risk for misuse and abuse, opioids are nonspecific analgesics that are not universally well tolerated; limiting or eliminating their use in favor of novel approaches is imperative. The use of opium and its compounds has been described since the Neolithic era; their ubiquity as analgesic agents have persisted into the 21st century. Individuals have varying responses to opioids with respect to analgesic efficacy and side effects/adverse events [35].

Recently, consideration has been given to non-pharmacological pain treatments. Several techniques have been proposed as an alternative to opioids, including acupuncture [36,37], hypnosis [38], transcutaneous electrical nerve stimulation [39,40], and auriculotherapy [41]. However, the effectiveness of these techniques remains to be established, as very few well-designed placebo-controlled studies have been conducted. Also, these complementary techniques require intensive training, which limits their acceptance.

Advancements in nano and quantum technology have led to an increasing number of medical applications [42,43,44,45,46,47,48,49,50]. In vivo indications are based on the use of nano-transporters, including nanoparticles [42], micelles, quantum dots [43], liposomes, nanofibers [42,43,44,45,46], and nano-scaffolds [47,48]. These modes of nano-transporters have allowed for the development of drug formulations with decreased drug toxicity, prolonged drug effectiveness, and in some cases, more specific delivery [49,50,51]. Examples include the use of liposomes to deliver amphotericin B and the use of chemotherapy to treat cancer [50]. Other in vivo applications are based on the development of nanomaterial-based biosensors and nano-biomarkers, which greatly improve image quality and the monitoring of physiological functions such as cardiac and nerve conduction [49] and intracellular metabolism [50]. The potential applications are considered endless.

Finally, advancements in nanotechnology have led to the development of nanocapacitor devices/patches. The use of this technology has been applied to control acute and chronic musculoskeletal pain and has demonstrated some degree of effectiveness.

## 2. Methods

Google and PubMed searches were conducted to identify studies, case reports, and reviews of medical nanotechnology applications with a special focus on acute and chronic pain. This search was based on the use of keywords like nanotechnology, nano and quantum technology, nanoparticles, micelles, quantum dots, liposomes, nanofibers, nano-scaffolds, acute and chronic pain, and analgesics. This review focuses on the role of nanotechnology in acute and chronic pain.

## 3. Results

### 3.1. Nanotechnology-Based Transporters 

The use of nanotechnology-based transporter for the treatment of pain has focused on liposome-based transporters. These formulations have been found to extend the duration of analgesia and reduce the toxicity of medications [51,52]. The first application was the basis of the development of DepoFoam™ a liposome-based formulation. It was combined with morphine for an epidural administration or DepoDur™ (Endo Pharmaceuticals Inc., Chadds Ford, PA, USA; Skye Pharma, Inc., San Diego, CA, USA). In a clinical trial involving patients undergoing THA, patients were randomized to receive a single dose of 15, 20, or 20 mg of DepoDur™ or placebo. All DepoDur™ dosages reduced intravenous patient-controlled fentanyl use (510 +/− 708 vs. 2091 +/− 1803 microg; *p* < 0.0001) and significantly improved pain control at rest through 48 h compared to the placebo (area under the curve (AUC) [0–48 h], *p* < 0.0005) [53]. In another randomized, double-blind study on lower abdominal surgery patients, single-dose of DepoDur™ provided superior pain control up to 48 h post-dose, both at rest and during activity, and significantly reduced intravenous patient-controlled fentanyl use compared to conventional epidural morphine [54].

DepoFoam™ was also used to develop a liposome-bupivacaine formulation for local injection either by surgeons or anesthesiologists. (Exparel™, Pacira Pharmacutical Inc., Parsippany-Troy Hill, NJ, USA) [55]. Although this technology allowed for the consistent release of the medication over an extended period [56], some publications failed to report superiority in terms of pain control or opioid consumption in postoperative pain management compared to conventional bupivacaine [57,58]. Exparel™ is currently FDA-approved for postsurgical local analgesia, bunionectomy, hemorrhoidectomy, interscalene nerve block, and adductor canal and sciatic nerve blocks [59].

The most recent use of a liposome preparation for the treatment of acute pain is a combination of bupivacaine and meloxicam (Zynrelef™, Heron Therapeutics, San Diego, CA, USA) [60]. In this formulation, meloxicam was found to increase the effectiveness of bupivacaine by maintaining the pH of the preparation when applied at the time of the wound closure. Zynrelef™ is reconstituted using a needle-free syringe and a luer-lock applicator, allowing for the application of the viscous solution at the level of the subcutaneous tissue and/or at the soft tissue incision level at the time of the wound closure. Zynrelef™ is approved as an agent allowing for the provision of 72 h of postoperative analgesia following open surgery. Zynrelef™, also referred to as HTX-011, is available in 60 mg/1.8, 200 mg/6 mg, 300 mg/9 mg, and 400 mg/12 mg of bupivacaine/meloxicam, respectively. Initially, it was approved by the FDA in 2021 to be used at the time of closure at the level of a medium incision following abdominal surgery, for open herniorrhaphy, total knee arthroplasty (TKA), and bunionectomy. This approval was based on positive results from phase III clinical studies conducted in these pain models. In trials conducted by Viscusi et al., the efficacy of bupivacaine/meloxicam PR against bupivacaine HCl and a placebo was compared in patients who underwent bunionectomy with osteotomy and internal fixation under regional anesthesia in the EPOCH 1 trial [61] and those who underwent unilateral open inguinal herniorrhaphy with mesh placement under general anesthesia in the EPOCH 2 trial [62]. In both trials, the use of Zynrelef™ was associated with significantly reduced postoperative pain and opioid consumption up to 72 h postoperatively compared to plain bupivacaine [62,63]. In 2024, the indications of Zynrelef™ were extended to include open shoulder and spine surgery. Contraindications included patients with established hypersensitivity to local anesthetics and non-steroidal anti-inflammatory drugs (NSAIDs), patients with a history of asthma, urticaria, or allergic reaction related to aspirin or other NSAIDs, patients receiving obstetric paracervical block anesthesia, and patients undergoing coronary bypass surgery. Recently, we reported that the use of Zynrelef™ was safe in combination with single nerve block performed prior to surgery. This conclusion was based on the administration of 115 bilateral single blocks with a total dose of 150 mg of bupivacaine or 200 mg of ropivacaine and the administration of unilateral blocks with 75 mg of bupivacaine or 100 mg of ropivacaine. Most of these blocks were quadratus lumborum blocks [64]. Prior to instillation, Zynrelef™ is reconstituted using a needle-free syringe and a luer-lock applicator, allowing for the application of the solution to the subcutaneous tissue at the time of closure. After reconstitution, Zynrelef™ is applied at either the peri-articular level and/or at the soft tissue incision level at the time of closure. Examples of studies conducted on liposome-based formulations (DepoDur™, Exparel™ and Zynrelef™) are presented in Table 1.

### 3.2. Nanotechnology-Based Devices/Patches

**Figure 1:**
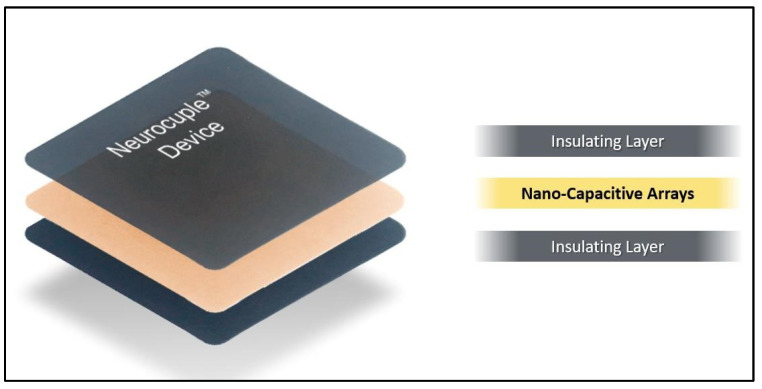
Nanotechnology pain device design.

In the past 10 years, nCap Medical LLC (Heber City, UT, USA) developed a nanocapacitor-based device/patch (referred to as the NeuroCuple™ device). Soon after, nCap Medical LLC also licensed the technology to Signal Relief technologies (Sandy, UT, USA) and Kailo Labs LLC (Sandy, UT, USA). This patented device/patch is constituted of three layers including a middle layer containing millions of nanocapacitors covered by two layers (Figure 1). The nanocapacitors operate using a method referred to as “capacitive coupling”. Practically, capacitive coupling allows for the transfer of electrical energy between two components without a direct electrical connection, utilizing the electric field and the magnetic field. The nanotechnology-based device/patch does not contain any chemicals, herbal medicines, or drugs and has no energy source. It can be reused over and over. To date, no side effects have been reported with the use of nanotechnology-based devices/patches.

In its current configuration, the NeuroCuple™ device/patch has a smooth side and a Velcro side. The smooth side is usually applied on the skin, whereas the Velcro side is used to secure the device/patch in place with tape or a piece of cloth. Because the NeuroCuple™ device/patch is not required to be applied on the skin, the Velcro side can be used to secure the device/patch on the outside of a piece of cloth or bandage. However, if a piece of cloth is chosen as the mode of securing the NeuroCuple™ device/patch, the cloth should be composed of a material that allows the device/patch to be kept in the same position to avoid any displacement of the device.

Prior to securing the NeuroCuple™ device/patch in place, it is critical to determine its optimal position. This is accomplished by first placing the NeuroCuple™ device/patch at the site of the pain. Within one to two minutes, the patient should experience some pain relief. If the effect is limited or no change in the pain level is observed, the next step is to move the NeuroCuple™ device/patch around the pain source to determine the patch’s optimal position before it is secured in place. Although the optimum effect of the NeuroCuple™ device/patch is usually achieved within minutes or an hour, sometimes the maximum therapeutic effect is observed hours or even one or two days after placement. The NeuroCuple™ device/patch can remain in place for hours or days. The best way to determine how long the NeuroCuple™ device/patch should remain in place is to remove it after one or two days and see if the pain continues. If pain is still present, the NeuroCuple™ device/patch can be placed at the same location for another day or more.

**Figure 2:**
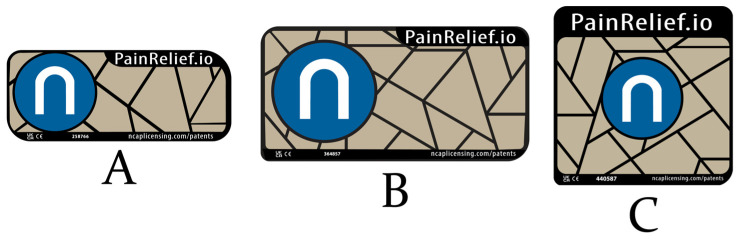
NeuroCuple™ device from nCap Medical LLC (Heber City, UT, USA).

The NeuroCuple™ device/patch comes in several shapes. Each shape was developed to optimally fit a specific part of the body. Thus, the small rectangular NeuroCuple™ device/patch is specially designed for migraine, wrist, and ankle pain (Figure 2A). To treat migraines, the NeuroCuple™ device/patch is placed at the site of the pain and can be secured using a head band. Larger rectangular and square NeuroCuple™ devices/patches are also available. These NeuroCuple™ devices/patches are best used to control back, shoulder, and knee pain (Figure 2B,C). Most evidence supports the use of the NeuroCuple™ device/patch to manage musculoskeletal acute and chronic pain; however, anecdotal reports also suggest that the nano-based technology may also effectively control abdominal and pelvic pain.

**Figure 3:**
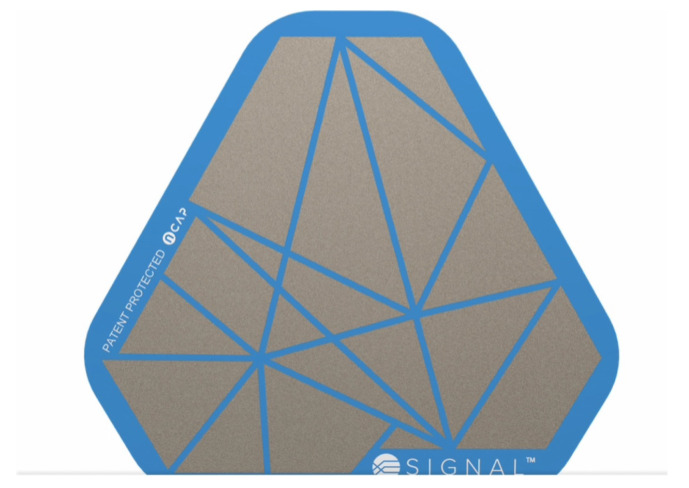
Nanotechnology pain device from Signal Relief™ technologies (Sandy, UT, USA).

**Figure 4:**
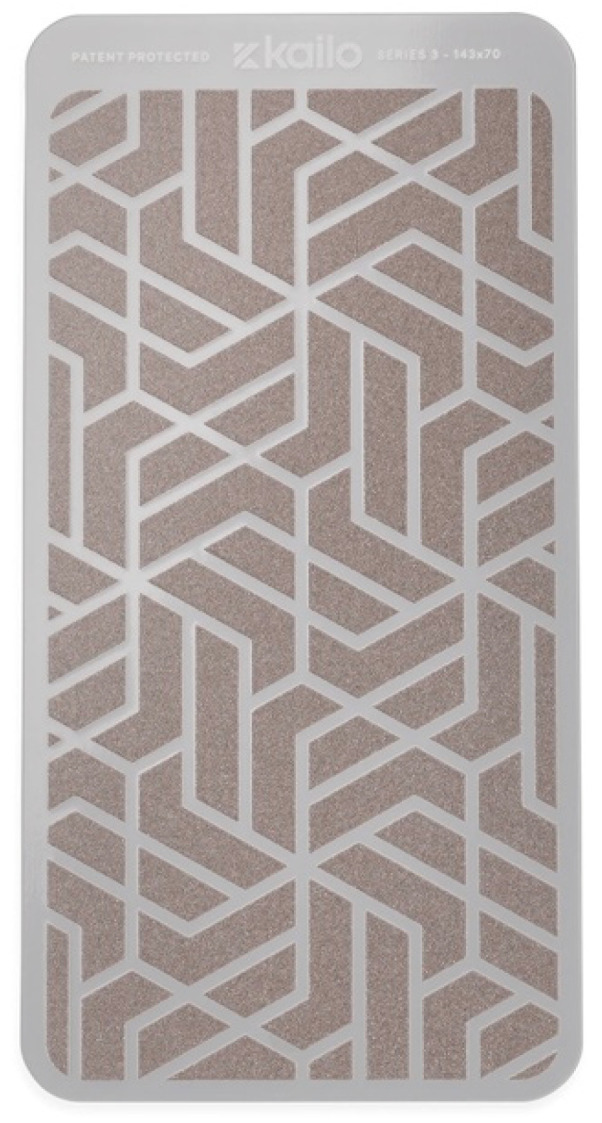
Nanotechnology pain device from Kailo™ Labs LLC (Sandy, UT, USA).

In addition to the original NeuroCuple™ devices/patches, two other versions are available: one developed and distributed by Signal Relief™ (Figure 3; Salt Lake City, UT, USA) and one by Kailo™ (Figure 4; Salt Lake City, UT, USA). Although, from a technical point of view, these nanotechnology-based device/patches are similar to the NeuroCuple™, they differ in shapes, colors, and modes of securement. The Signal Relief™ device/patch is triangular, comes in either blue or white, and it is available in two sizes, 4.5″ and 1.5″ (Figure 3). The Kailo™ device also contains two conductive elements, copper and silver, visible on the surface of the patch (Figure 4). However, both companies’ devices/patches use a double adhesive transparent liner to attach the device/patch to the skin. One of the adhesive sides is attached to the device/patch, while the other adhesive side is used to secure the device/patch on the skin.

**Figure 5:**
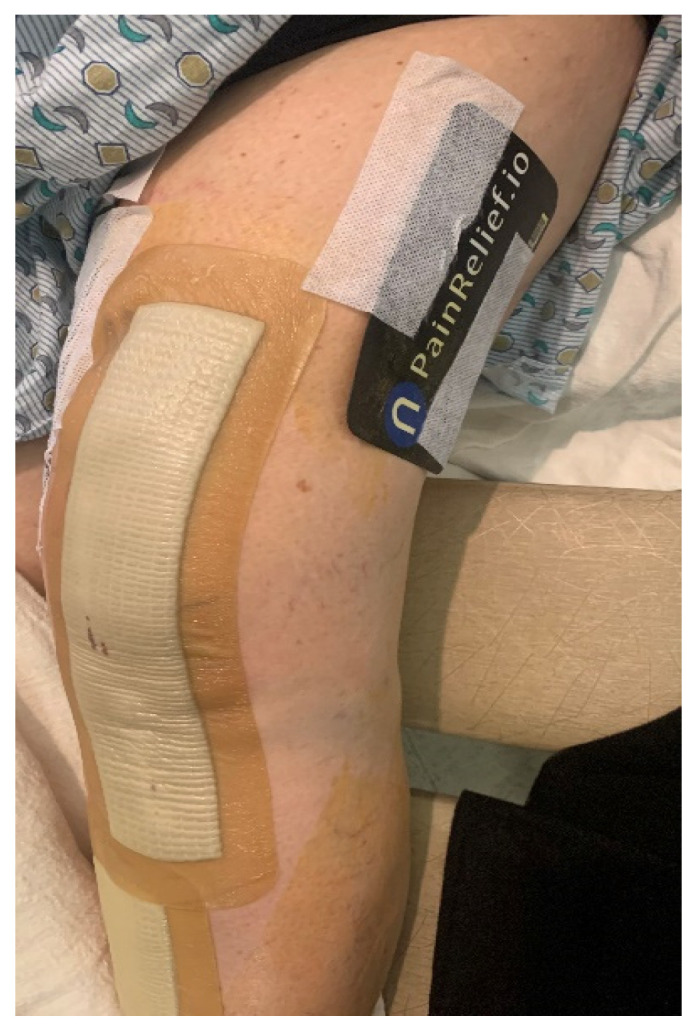
Two NeuroCuple™ devices placed on each side of the thigh to control pain following total knee arthroplasty.

Evidence supporting the use of nano-based technology to treat acute and chronic pain is based on two studies. The first was a multicentric study conducted in three US centers by Gudin et al. that included 128 patients (89 females, 39 males), mean age 47 years old with chronic pain in one body location for at least one year. The pain was related to arthritis (23.4%), neuropathy or radiculopathy (30.5%), or myofascial or musculoskeletal pain or spasm at the level of the hands, feet, hips, knees, neck, shoulders, or back (46.1%). The goal of this prospective, institutional review board (IRB)-approved observational study was to assess the analgesic properties of the Kailo™ nanocapacitor-based device/patch (Figure 4; Salt Lake City, UT, USA, referred to as the Kailo™ Pain Patch). The primary end point was the effects of the application of the Kailo™ Pain Patch on pain intensity, measured using the Brief Pain Inventory questionnaire. Secondary end points included self-perceived pain relief from analgesics, patient satisfaction, quality of life, and ability to return to normal activities. Data were collected at baseline, on day 14, and on day 30. The patients were asked to wear the Kailo™ Pain Patch as long as necessary and for up to 30 days. Twenty patients were included as controls and crossed over after 14 days [65]. At 30 days, the use of the Kailo™ Pain Patch resulted in a 61% decrease in pain severity and interference, whereas a 23% increase in pain severity and a 57% increase in pain interference were recorded in the control group. Also, in the treatment group, 91% of patients reported “less” or “a lot less” use of oral analgesics and 86% of patients were very/extremely satisfied. Data also showed quality of life improvements in mood, relations with other people, sleep, walking ability, and the enjoyment of life. Most patients indicated that they wore the device/patch all the time during the study period. This may explain why between day 14 and day 30 the pain severity dropped by an additional 35% (40% vs. 61%). Although the study was observational, data supported the benefit of the nanotechnology-based device/patch for the treatment of chronic pain. However, it is clear that because of the observational nature of the study and the subjective-based design, additional studies are required to confirm these preliminary results. We also tested the NeuroCuple™ device/patch analgesic properties on patients undergoing primary and unilateral TKA and THA. Prior to any patient enrollment, this prospective, randomized study was approved by the IRB and registered with clinicaltrials.gov. Patients who agreed to participate gave informed consent; those who qualified were randomly distributed to either a standard of care group or a group who received standard of care plus two NeuroCuple™ devices/patches. The NeuroCuple™ devices/patches were placed above the knee or on the hip or each side of the thigh after the patient was situated in the recovery room (Figure 5) [66]. Because mood disorders have been established to significantly affect pain intensity and opioid consumption [67,68,69], only patients without evidence of anxiety, depression, sleep disorders, and catastrophizing were randomized. The primary end points were pain and opioid consumption. The secondary end points were time to discharge from the recovery room and the hospital, PROMIS scores [70], catastrophizing scores [71,72], and patient satisfaction. A total of 163 patients were screened, and 69 patients were randomized. We demonstrated that the use of NeuroCuple™ devices/patches reduced postoperative pain at rest by 34% (using the AUC between day 1 and day 30; *p* = 0.018), decreased pain with movement by 18% (using the AUC between day 1 and day 30, *p* = 0.12), and decreased the number of opioid refills requested by patients after discharge from the hospital by 52%. Table 2 present a summary of the 2 studies illustrating the use on nanocapacitor-based technologies for the treatment of chronic and acute pain.

## 4. Discussion

Besides demonstrating that the use of liposome extends the duration of analgesia, evidence suggests that the use of these nano-transporters can also decrease drugs toxicity. This principle has been applied to the use of drugs with known toxicity like amphotericin B [49]. It would be potentially beneficial to use nanotechnology-based to decrease the cardiac, gastrointestinal, renal and respiratory toxicity of opioids and anti-inflammatory drugs administered orally [74,75,76,77,78,79]. One possible barrier may be the fact that there is no nanotechnology-based oral formulation available. 

Although the indications of nanotechnology-based transporters seem to be endless, it is difficult to assess the future of nanotechnology-based transporters in pain. Today, the use of Depodur™ is limited. The use of Exparel by surgeons and anesthesiologists is much more important despite the relative controversy related to its long-lasting effects. Zynrelef™ introduction is too recent to assess its future use. However, a lidocaine liposome as a gel formulation is being developed. Data reported in animals indicate that this preparation may be interesting in human [80].

**Figure 6:**
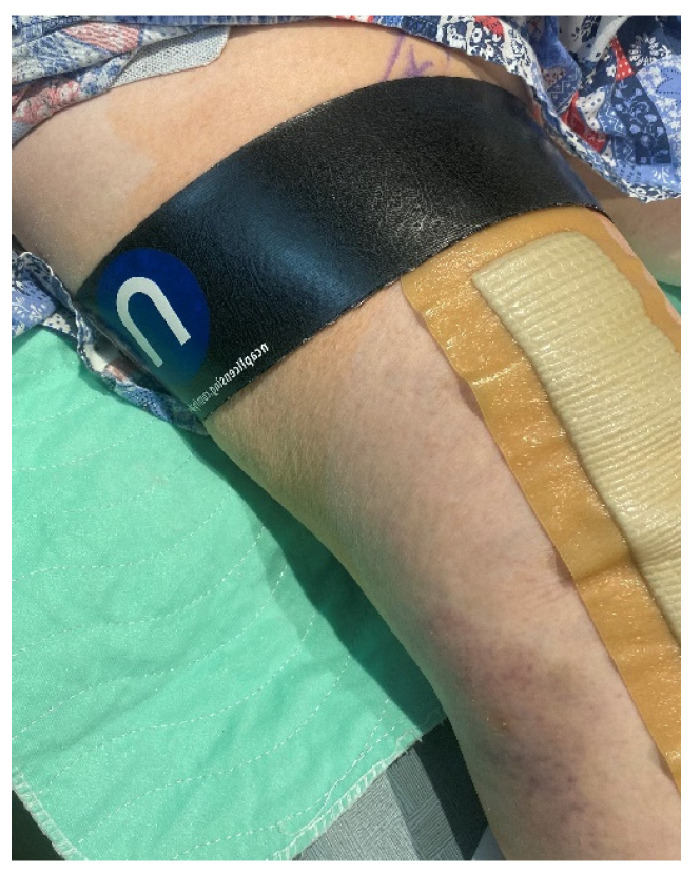
One NeuroCuple™ device placed above the knee to control pain following total knee arthroplasty.

After conducting our randomized standard of care vs standard of care plus the NeuroCuple™ device in patients undergoing primary unilateral TKA and THA, it became apparent that another study was required to confirm our preliminary data and that the design of the study needed to include a NeuroCuple™ device allowing to cover a larger surface area and to study the response obtained with an active NeuroCuple™ device vs. a placebo NeuroCuple™ device. Such a device was developed. It is presented in Figure 6. Soon after an active NeuroCuple™ device was developed, a prototype was applied on a patient’s thigh following a primary and unilateral TKA. The patient reported no use of opioid. This experience was published as a case report [73].

The mechanism of action of the nanocapacitor device/patch is still under investigation. Gudin et al. [66] claimed that the observed benefit of the Kailo™ patch was the result of a central effect. In contrast, we proposed that the mechanism of action of the NeuroCuple™ device was peripheral, suggesting that the nanocapacitors contained within the device represents as an alternative to the biological capacitors that are locally destroyed in case of a local trauma. Thus, in physiological conditions, it is established that cellular membranes play the role of biological capacitors which allows for electrical equilibrium. In trauma, the membrane is destroyed, resulting in an accumulation of electrons, a decrease in pH, and an accumulation of fluid, which generates local inflammation and pain. When applied locally, as an invitro capacitor, the NeuroCuple™ allows a redistribution of the local excess of electrons. This normalizes the local pH allowing inflammation and pain to be reduced [81,82,83,84,85]. Evidence supporting this concept is illustrated by measuring the local electromagnetic field changes associated with the local application of the NeuroCuple™. When the NEUROCUPLE device was applied just above a patient’s knee following TKA, capacitance increased from 35 pF to 455–474 pF using the Mastech MS8040 (Mastech Digital Inc., Pittsburgh, PA, USA). 

Most evidence supports the use of the nano-capacitors devices/patches distributed by nCap Medical, Sign Relief Technologies and Kailo Labs LLC for the management of musculoskeletal acute and chronic pain; however, anecdotal reports also suggest that the use of nanocapacitors may also be effective to manage migraine, fibromyalgia and abdominal and pelvic pain. There is no doubt that nanocapacitor devices/patches represent a fascinating technology, but their limits need to be tested, because beside their indications for the management of pain, premilitary data also suggests that the technology may have interesting indications in the treatment of cancer.

## 5. Conclusions

Nanotechnology offers promising options for the treatment of pain either as drug transporters or as capacitor device/patch. As a drug transporter-based formulations for morphine, bupivacaine and lidocaine nanotechnology allows for extending the duration of these drugs, a reduction in the effective analgesic dose, therefore reducing potential drug toxicity. As nanocapacitor-based technology it potentially represents an effective non-pharmacology approach. In addition, compared to other complementary techniques the use of nanocapacitor based device/patch don’t require training. However, additional research is required to establish the usefulness of this technology for the treatment of chronic and acute pain.

## Figures and Tables

**Table 1:** Examples of studies conducted on liposome-based formulations.

Keyword	Study	Authors	# Patients	Treatment vs. Comparison	Results
DepoDur™	Forty-Eight Hours of Postoperative Pain Relief after Total Hip Arthroplasty (THA)	Viscusi et al. (2005) [53]	200	15, 20, or 25 mg DepoDur™ vs. saline	All dosages reduced fentanyl use (510 ± 708 vs. 2091 ± 1803 microg) and delayed time to first dose of fentanyl (21.3 vs. 3.6 h).
	A Comparison of DepoDur™ to Standard Epidural Morphine for Pain Relief After Lower Abdominal Surgery	Gambling et al. (2005) [54]	541	5, 10, 15, 20, or 25 mg of single-dose DepoDur™ vs. 5 mg of standard epidural morphine sulfate	Patients who received 10, 20, or 25 mg single doses used significantly less intravenous fentanyl through 48 h postoperatively.
Exparel™	Exparel™ for Postoperative Pain Management: A Comprehensive Review	Kaye et al. (2020) [56]	Review	Liposomal bupivacaine vs. standard local anesthetic or placebo	Liposomal bupivacaine provided prolonged analgesia and opioid-sparing effect compared to placebo.
	The Efficacy of Liposomal Bupivacaine for Opioid and Pain Reduction: A Systematic Review of Randomized Clinical Trials	Ji et al. (2021) [57]	6770	Liposomal bupivacaine vs. other active agents or placebo	Out of 77 identified trials, liposomal bupivacaine did not demonstrate better pain relief in 74.58% of trials compared to other active agents or placebo. It did not show reduction in opioid consumption in 85.71% of trials.
	Liposomal Bupivacaine in Adductor Canal Block (ACB) before Total Knee Arthroplasty (TKA)	Malige et al. (2022) [58]	100	Liposomal bupivacaine 20 cc with 5 cc of 0.5% bupivacaine in ACB and 20 cc of 0.2% ropivacaine in iPACK block vs. 25 cc of 0.2% ropivacaine in ACB and 20 cc of 0.2% ropivacaine in iPACK block	Subjects receiving liposomal bupivacaine had shorter hospital stays compared to the ropivacaine group (36.3 vs. 49.7 h). Liposomal bupivacaine decreased pain and reduced inpatient opioid consumption compared to ropivacaine group (40.9 vs. 47.3 MME/d).
Zynrelef™	HTX-011 Reduced Pain Intensity and Opioid Consumption versus Bupivacaine HCl in Bunionectomy: Phase III Results from the Randomized EPOCH 1 Study Bupivacaine/Meloxicam Prolonged Release: A Review in Postoperative Pain	Viscusi et al. (2019) [61]	412 subjects undergoing bunionectomy	Bupivacaine/meloxicam 60/1.8 mg vs. bupivacaine HCl 0.5% 50 mg vs. saline placebo 2.1	Bupivacaine/meloxicam combination reduced pain intensity by 27% vs. saline placebo and 18% compared to bupivacaine. Opioid consumption was reduced by 37% in bupivacaine/meloxicam group vs. saline placebo and 25% vs. bupivacaine group.
	Study Bupivacaine/Meloxicam Prolonged Release: A Review in Postoperative Pain	Blair et al. (2021) [63]	Review of two randomized controlled trials on bunionectomy and herniorrhaphy	Bupivacaine/meloxicam vs. bupivacaine HCl vs. saline placebo	As part of non-opioid multimodal analgesia, bupivacaine/meloxicam improved pain control and reduced need for opioids in postoperative period.
	Safety and Efficacy of Zynrelef™ in Combination with a Single Unilateral or Bilateral Nerve Block Performed Prior to Surgery	Goel et al. (2023) [64]	184	All received bupivacaine/meloxicam	No symptoms suggestive of local anesthetic toxicity were reported. Use of combination was associated with 50% reduction in number of patients filling their opioid prescriptions.

**Table 2:** Example of studies conducted on nanocapacitor-based technologies.

Keyword	Study	Authors	# Patients	Treatment vs. Comparison	Results
Nanocapacitor device/patch	Use of Nanocapacitors for the Control of Chronic Pain Observational study	Gudin et al. (2022) [65]	148	Kailo™ Pain Relieving Patch vs. no patch	Over 30 days, Brief Pain Inventory (BPI) severity scores in the treatment group decreased by 61%, and the mean BPI interference score decreased by 61%.In contrast, in the control group, the BPI severity score increased by 23%, and the BPI interference score increased by 58%.
	Role of the NeuroCuple™ Device for the Postoperative Pain Management of Patients Undergoing Unilateral Primary Total Knee and Hip Arthroplasty: A Pilot Prospective, Randomized, Open-Label Study	Chelly et al. (2023) [66]	69	NeuroCuple device/patch vs. no device/patch	Patients who received the device/patch experienced lower pain levels at rest during postoperative days 1–3, with a 34% reduction in postoperative pain compared to patients without the device/patch, the standard of care. The use of the device/patch reduced the number of opioid refills by 52%.
	Use of Nanotechnology as an Alternative to Opioids for Postoperative Pain Management Following TKA Case report	Chelly et al. (2023) [73]	1		The patient did not require any opioids postoperatively with the use of the nanocapacitor-based device/patch.
